# Big data, big lies?

**DOI:** 10.1016/j.xfre.2022.11.006

**Published:** 2022-11-16

**Authors:** Richard J. Paulson

**Affiliations:** Division of Reproductive Endocrinology and Infertility, Department of Obstetrics and Gynecology, University of Southern California, Los Angeles, California

**Keywords:** Statistics, registries, big data

One of the consequences of the “connected” world has been the establishment of large computerized databases. These databases are like goldmines of data, and they are often mined for “Big Data” analyses. In the world of in vitro fertilization (IVF), the Society for Assisted Reproductive Technology database collects information from participating North American IVF clinics, while the Human Fertility and Embryology Authority database does so for clinics in the United Kingdom, but there are many others.

Large IVF databases are good for providing estimates of the total volume of cases, the overall number of live births, or the geographic distribution of clinics among the participating reporting centers. They are also excellent for observing trends in treatment over time, such as the change in the usage of embryo cryopreservation, intracytoplasmic sperm injection, or preimplantation genetic testing for aneuploidy. However, these databases are often queried for associations between various treatment modalities and subsequent pregnancy outcomes; this is where we must be cautious not to infer causation from observed correlations.

All retrospective data analyses are confounded by the nonrandom assignment of patients to different treatment categories. This treatment choice may be selected by the patients themselves (such as socioeconomic factors driving the choice of therapy) or by the treating physicians (different procedures chosen for cases that are expected to be less or more complex). There are patient characteristics that may affect the outcome, such as age or body mass index (BMI); these are often recorded in the database and can generally be accounted for. In contrast, subtle differences in the choice of treatment because of patient or physician choice are invisible to the data analysis. Furthermore, the large number of cases available for analysis in a national database amplifies small differences and imbues them with statistical significance.

Let us consider the example of gonadotropin dose and IVF pregnancy outcome. Physicians choose gonadotropin doses based on the anticipated response to ovarian stimulation. Patients who are expected to have a low response are generally given higher doses of stimulation. In a retrospective analysis of data extracted from a large database, we may note that higher gonadotropin doses are associated with poorer outcomes. It would be wrong to infer that the higher gonadotropin dose resulted in poor oocyte quality or that strong stimulation somehow disrupted endometrial receptivity. It might just be that patients who are expected to be poor responders are administered higher doses of gonadotropins, and those same patients also generally have worse outcomes.

Perceived patient characteristics may also drive the choice of endometrial preparation for frozen embryo transfer. If a patient undergoing a frozen embryo transfer has irregular cycles, a higher BMI, or other features of polycystic ovarian syndrome, she is more likely to be treated with an artificial cycle of exogenous steroids. In contrast, a patient with regular menses and a lower BMI is more likely to undergo frozen embryo transfer in a monitored ovulatory cycle. If the former patient’s physical characteristics predispose her to a diagnosis of hypertensive disorder of pregnancy, a retrospective analysis will reveal an apparent association between the artificial cycle and hypertensive disorder of pregnancy, even if the actual endometrial preparation or the presence of a corpus luteum had no physiological effect.

In a database that collects data from hundreds of clinics, outcome data may be confounded by the choice made by individual clinics or physicians to offer or not to offer a particular treatment. For example, clinics may have different age cut-offs for prospective egg donation recipients. Women over 50 years of age often struggle to find a clinic willing to perform an embryo transfer and, thus, may end up at a clinic where recipients experience lower pregnancy rates. Even if every recipient of egg donation experiences the same pregnancy rate as every other recipient in any given clinic, regardless of age, the (hypothetical) preponderance of older recipients in clinics with lower pregnancy rates will make the overall rate of success in the database appear to be lower in these recipients.

These are just a few examples of prior retrospective studies with data derived from large registries. There are many other examples of associations between treatments and outcomes attributed to factors other than actual physiologic causation. This is why the establishment of real treatment benefits requires multiple cohort studies, physiologic plausibility, and, hopefully, at least one randomized clinical trial. Large databases are designed to report outcomes associated with treatment. However, when comparing outcomes associated with different treatment options, retrospective analyses are useful only for generating hypotheses, not for providing evidence for causality. Let us appreciate Big Data, but let us not be led to conclude Big Lies.

